# Consulting the Oracle: A Delphi study for determining parameters for a mental health user profile and personalization strategy for an online service to aid grieving older adults

**DOI:** 10.1016/j.invent.2022.100534

**Published:** 2022-04-05

**Authors:** Lena Brandl, Miriam Cabrita, Jeannette Brodbeck, Dirk Heylen, Lex van Velsen

**Affiliations:** a Roessingh Research and Development, P.O. Box 1212, Enschede, the Netherlands; b University of Twente, Faculty of Electrical Engineering, Mathematics and Computer Science, Department of Human Media Interaction, P.O. Box 217, 7500AE Enschede, the Netherlands; c University of Twente, Faculty of Electrical Engineering, Mathematics and Computer Science, Department of Biomedical Signals and Systems, P.O. Box 217, 7500AE Enschede, the Netherlands; d Institute for Consulting, Coaching and Social Management, FHNW School of Social Work, Riggenbachstrasse 16, 4600 Olten, Switzerland; e Universität Bern, Institut für Psychologie, Fabrikstrasse 8, 3012 Bern, Switzerland

**Keywords:** Mental e-health, Tailoring, Personalization, Mental user profile, Older adults, Grief

## Abstract

While much effort has been devoted to the development of mental e-health interventions, the tailoring of these applications to user characteristics and needs is a comparatively novel field of research. The premise of personalizing mental e-health interventions is that personalization increases user motivation and (thereby) mitigates intervention dropout and enhances clinical effectiveness. In this study, we selected user profile parameters for personalizing a mental e-health intervention for older adults who lost their spouse. We conducted a three-round Delphi study involving an international and interdisciplinary expert panel (*N* = 16) with two objectives. The first aim was to elicit adaptation strategies that can be used to dynamically readjust the intervention to the user's needs. The second aim was to identify a set of meaningful indicators for monitoring the user from within the grief intervention to escalate from self-help to blended care, whenever advisable. This Delphi study used as starting point an evaluated, text-based grief intervention composed of ten modules, including psychoeducation about grief and cognitive-behavioral exercises to support the user in adjusting their lives after bereavement. Every user follows this grief intervention in a linear fashion from beginning to end. The resulting conceptual adaptation model encompasses *dynamic* adjustments, as well as one-time adjustments performed at the *initialization* of the service. On the level of the application structure, the adaptations affect *when which* topic module is presented to the user. The adaptations further provide strategies for adjusting the text-based content of individual intervention modules dependent on user characteristics and for selecting appropriate reactions to user input. Eighteen monitoring parameters were elicited and grouped into four categories: *clinical*, *behavioral/emotional*, *interactive*, and *external*. Parameters that were perceived as most urgent to attend to for escalation were *Suicidality*, *Self-destructive behavior*, *Client-initiated escalation*, *Unresponsiveness* and *(Complicated) Grief symptoms*.

## Highlights

• Rates tailoring strategies for an online grief service for older mourners who lost their spouse via a Delphi study.
• Presents a tailoring model including adaptations that impact the selection, order, and approach of intervention topics.
• Tailoring an online grief service can be one-time as well as consisting of dynamic adjustments.
• Ranks user parameters to inform a mental health user profile.
• The ranking can inform user profiles aiming to guide users of an online grief service to offline support.

## 1 Introduction

The loss of a spouse is a frequent occurrence in later life. While most bereaved adults successfully process the loss and continue to lead a normal life, some (about 9% of the bereaved population according to a recent prevalence study (Wilson et al., 2020)) have difficulties overcoming bereavement and develop complicated grief. Complicated grief in adults is a condition where severe grief symptoms occur longer than six months after bereavement and frequently results in a multitude of mental and physical problems, such as depression, loneliness, cardiovascular problems and, in extreme cases, suicidal tendencies (Molina et al., 2019). Internet-based (mental e-health) interventions have been shown to be effective in treating mental illnesses, including complicated grief (Brodbeck et al., 2017; Carlbring et al., 2018; Eisma et al., 2015; Wagner et al., 2006). Benefits of e-health interventions compared to face-to-face therapy are low threshold accessibility, flexible usage at a self-determined pace, and lower costs (Schröder et al., 2016). Internet-based interventions often combine a web-based self-help program and minimal, but regular therapist contact. The inclusion of regular professional guidance has been shown to improve adherence and clinical outcomes compared to standalone self-help programs (Baumeister et al., 2014), but less is known about how this support needs to be delivered. Support on-demand, where contact is initiated by the client and focused on the specific needs they have at that moment, has been suggested to optimize the incorporation of therapists in blended internet interventions (Dahlin et al., 2020; Oromendia et al., 2016). Here, we consider a complementary strategy for support on-demand: automated monitoring of the user's symptoms and situation while they use the intervention with the purpose of timely escalation if they end up needing more intensive professional support. This escalation could suggest to schedule a telephone or face-to-face meeting with a professional, if advisable. For this, a user profile is needed consisting of relevant indicators of the user's ability to continue working on the intervention by themselves, without professional intervention. These indicators should be optimized for the specific type of mental e-health intervention. Finally, a decision algorithm that combines these indicators into actionable advice needs to be developed.

A second consideration about mental e-health interventions is that a client's journey through mental illness is inherently personal. Indeed, therapists personalize face-to-face therapy readily by skipping or modifying therapeutic protocols to adapt to the client's needs and preferences and to increase the client's adherence to therapy (van Dooren et al., 2020). Lack of adherence, i.e., intervention dropout, has been recognized as a core challenge for e-health interventions and personalization is a primary strategy for mitigating dropout (Burley et al., 2020; Eysenbach, 2005). Co-design of personalization strategies together with (clinical) professionals and end-users is essential, inherently multidisciplinary and challenging (Pagliari, 2007; van Dooren et al., 2020). Different expertises (e.g., therapists, designers, software developers) and disciplines (e.g., cognitive-behavioral, psychoanalytical therapists) come together, sometimes in multicultural settings accompanied with language barriers. In this paper, we demonstrate the administration of the Delphi method (Okoli and Pawlowski, 2004; Schmidt, 1997) to early-stage personalization research for mental e-health interventions, involving an multidisciplinary expert panel working in four European countries.

This study has two objectives. First, we aim to find a personalization strategy for an internet-based grief intervention for older adults who lost their spouse. Second, we strive to determine a set of indicators that can be used to monitor the user for the purpose of delivering professional support on-demand. In particular, we will answer the following research questions:

RQ1 What is a suitable personalization strategy for tailoring an online grief intervention for older adults who lost their spouse to the characteristics and needs of the individual user?

RQ2 Which user parameters should be included in a user profile of an online grief intervention for older adults with the purpose of delivering professional support on-demand if they end up needing more intensive support?

We use the Delphi method (Okoli and Pawlowski, 2004; Schmidt, 1997) to consult an expert panel of clinical professionals regarding how they personalize their therapy and combine their knowledge with the knowledge of e-health experts to yield actionable ideas for adaptations and indicators for monitoring. The Delphi method is commonly employed to reach agreement when literature is inconclusive or incomplete (Schmidt, 1997; Straat et al., 2020) and it is suitable for administration in multidisciplinary, multilanguage expert panels as the researcher can facilitate the group communication process.

## 2 Background

### 2.1 Personalized (mental) e-health

In recent years, the personalization of mental e-health has received much interest. A variety of personalized mental e-health interventions have been developed and evaluated, including web-based interventions for treating anxiety (Carlbring et al., 2011) and depression (Johansson et al., 2012) and mobile interventions, such as Woebot, targeted at young adults with symptoms of depression and anxiety (Fitzpatrick et al., 2017). Nevertheless, there is no unified definition of personalized e-health and the term *tailoring* is often used interchangeably (e.g., Lustria et al., 2013; Ryan et al., 2019). Noar (Noar et al., 2011) explains the *tailoring process* as follows: characteristics of the person are gathered, either by another person or self-administered, and represent the *input* for the tailoring process. The input is processed either by a human or a computer that uses an algorithm to select content from an expert-developed database, such as texts, images, recommendations, and intervention messages. This is called the *tailoring process*. Finally, the tailored material (*output*) is optimized for the delivery mode at hand and presented to the individual. According to Noar, the premise behind personalization is that it increases the relevance of the intervention for the individual, who is subsequently more likely to cognitively process and to adhere to the presented health advice.

Personalization strategies that have been employed to increase the relevance of mental e-health interventions vary greatly in complexity, ranging from inserting the individual's name in the intervention, to adapting content based on user characteristics (Morrison, 2015), including clinical characteristics. Berger, Boettcher and Casper (Berger et al., 2014) use cut-off scores from disorder-specific self-report questionnaires to tailor the selection of treatment content in an intervention targeted at several anxiety disorders. Carlbring, Maurin, Törngren et al. (Carlbring et al., 2018) compose individually-tailored anxiety intervention programs targeted at comorbidities on the basis of structured clinical interviews for DSM disorders (SCID) prior to the start of the intervention. Other personalization strategies include user preferences and demographics (e.g., relationship status, status of employment) to optimize the relevance of scenarios with which the user practices healthy thinking patterns (Burley et al., 2020).

In conclusion, personalized mental e-health is a vast research discipline, with no clear guidelines regarding the development of personalization strategies. Nevertheless, it appears that effective personalized interventions combine a) an evidence-based therapeutic foundation, b) careful selection of user characteristics to base the tailoring process on, and c) an expert-informed choice of therapeutic content that is suitable for personalization (van Dooren et al., 2020).

### 2.2 Case study of an online grief intervention

Our efforts to unravel parameters for automated monitoring and a personalization strategy are evidence-based. They draw on a text-based online intervention for older adults who have lost their spouse due to bereavement or divorce, called LIVIA (Brodbeck et al., 2019). The therapeutic content of the intervention was designed by professionals based on theoretical models of grief and components of cognitive-behavioral therapy for treating complicated grief (Brodbeck et al., 2017). The intervention is divided in ten modules that the user works through in their own pace. Modules consist of psychoeducation about the grief trajectory and cognitive-behavioral writing exercises to support the user in adjusting their lives after bereavement. For instance, the first module -*Psychoeducation* - consists of information about grief reactions, emotional reactions after bereavement and the (clinical) treatment of grief. The second module -*Assessment of the current situation* - reflects on the user's emotional reactions after the loss, changes in life since the loss and obstacles for positive adaptation. The intervention further encompasses topics related to self-care, the identification of changes in the daily routine since the loss and unresolved issues in the relationship to the lost spouse. It concludes with writing a farewell letter. The results of this study will inform a re-design of the intervention as part of the AAL LEAVES project (van Velsen et al., 2020). The re-design will enable the resulting intervention, called *LEAVES* in the remainder of this paper, to a) monitor a users' situation, with the goal to deliver support on-demand in the form of telephone calls or face-to-face meetings, whenever advisable and b) to tailor the therapeutic content based on user characteristics and needs to enhance user adherence and thereby clinical effectiveness. Finally, as part of the re-design and to make the intervention more interactive, the text-based content will be re-written into a dialogue format and delivered by an embodied conversational agent (ECA).

## 3 Method

### 3.1 Study design

**Fig. 1:**
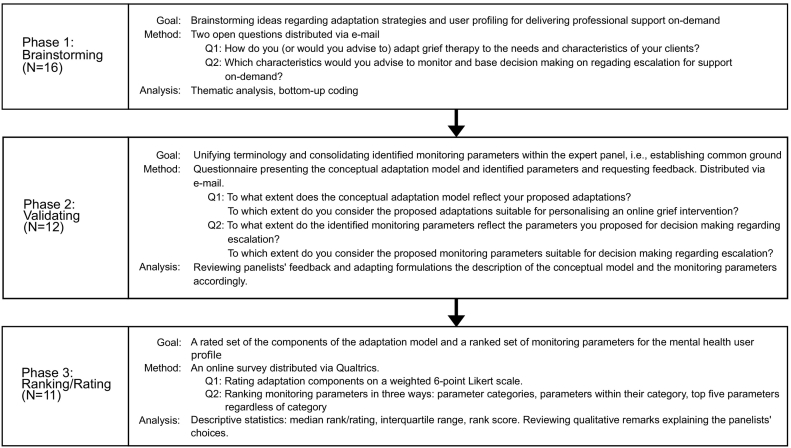
Overview of the three-phase Delphi design.

A Delphi study is a systematic polling of the opinions of an expert panel, knowledgeable on a specialist topic through an iterative survey, usually in an attempt to reach group consensus on a given topic (Schmidt, 1997). A three-phase Delphi study involving 16 experts was conducted between June and December 2020. Fig. 1 shows the process of the Delphi study. The design followed the three-phase design of Okoli and Pawlowski (Okoli and Pawlowski, 2004) with two notable deviations. First, we distributed three instead of four questionnaires in total. Okoli and Pawlowski administered two questionnaires in the first, the *brainstorming phase* and used the second phase of their study to narrow down the number of items they extracted from the first phase. We devoted the first questionnaire to brainstorming and the second to validating the items we elicited in the first questionnaire, thereby leaving out the step of narrowing down the number of items, for two reasons. First, the multidisciplinary nature of the conceptual adaptation model required our experts to transfer their discipline knowledge. We considered a validation of the model essential for establishing common ground for the final phase of the Delphi study, the *ranking*. Second, a common reason for narrowing down the amount of items is that it tremendously decreases the cognitive load of ranking tasks (Alwin and Krosnick, 1985). In our case, there was no need to narrow down the number of items for ranking because we designed the ranking in such a way that its cognitive load was minimized.

In the third round - the *ranking* - we did not strive for consensus, which constitutes a second deviation from Okoli and Pawlowski. Reaching consensus is often regarded as a necessary criterion for finalizing data collection in a Delphi study and this has been criticized before for its inappropriateness and artificiality of results (Dajani et al., 1979; von der Gracht, 2012). We asked our expert panel to rank the extracted adaptation strategies and user parameters for the monitoring in terms of maximal effect for the intervention (adaptation strategies) or clinical relevance (user parameters).

### 3.2 Participants

Experts were recruited via professional networks and by searching on the web. Search criteria were a) the expertise of the experts (grief, e-health or both) and b) the country where they work. Since the results of this study will inform the design of the LEAVES grief intervention that is targeted at Dutch, Portuguese and Swiss older mourners, we recruited experts originating from these three countries. The recruited experts worked as researchers in the field of grief or e-health or they practiced grief therapy, or both. Prior to the start of the study, all participants provided written informed consent to partake in the study and to reveal their identity to the rest of the expert panel after data collection had been finalized. Following (Miaskiewicz and Kozar, 2011; Okoli and Pawlowski, 2004; West, 2011), we collected data electronically, via e-mail, so as to ensure timely data collection and analysis. This was of importance to allow for conducting the different rounds in the study within a timeframe that was acceptable to the participants. Indeed, panel fatigue has been recognized as a challenge in Delphi studies (Hasson et al., 2000; Schmidt, 1997), as has the generation of large amounts of data that the research team has to process between questionnaire rounds (Green et al., 1999; West, 2011). Implementing a questionnaire with open-ended questions and recommending that each panel member provides a specific number of items, as suggested by (Schmidt, 1997; West, 2011), allowed our panel members to generate ideas while allowing the research team to process their input within a reasonable timeframe. Table 1 provides an overview of the recruited expert panel. Between phase one and three of the study, we experienced five dropouts. As a result, the second questionnaire was administered among twelve experts and the final questionnaire among eleven experts. Reasons for discontinuing their participation in the study were a) lack of time (*n* = 1) b) personal circumstances (*n* = 2) and c) lack of confidence in their responses (*n* = 2).

**Table 1:** Descriptives of the recruited expert panel.

Participant #	Gender (F = 10, M = 6)	Country (GER = 2, NL = 7, CH = 5, PT = 4)	Experience	Expertise
1	F	GER	Research	Grief
2	F	GER	Research/Clinical	Grief
3	F	NL	Research	Grief
4	M	NL	Research	e-health
5	M	NL	Research	e-health
6	F	NL	Research/Clinical	Grief
7	M	NL	Research	Grief
8	F	NL	Research/Clinical	Grief
9	F	NL	Research	e-health
10	M	CH	Research	Grief
11	F	CH	Research/Clinical	Grief/e-health
12	M	CH	Research	Grief/e-health
13	F	PT	Clinical	Grief
14	M	PT	Clinical	Grief
15	F	PT	Research/Clinical	Grief
16	F	PT	Clinical	Grief

## 4 Delphi round 1

### 4.1 Method

The first questionnaire served the purpose of brainstorming user parameters that are important for making well-founded decisions regarding monitoring users from within the grief intervention and about adaptations that can be performed to make the LEAVES intervention more personal. Experts were asked to list five characteristics of (older) mourners that they consider important to attend to for monitoring based on their clinical and/or research expertise. A second question asked the experts to consider how they adapt grief therapy to their own clients or how they would adapt therapy in practice based on their discipline knowledge to meet the individual needs of clients. Since the second question was quite abstract, an illustrative example was given for a plausible adaptation of the LEAVES service. The example was based on the influential Dual Process Model of Bereavement (DPM) (Schut and Stroebe, 1999; Stroebe and Schut, 2010), which considers oscillating between focusing on the loss and on restoration essential for the recovery of the bereaved person. We suggested that one way to adapt the service to the needs of the user was to monitor their orientation and to nudge the user to either orientation if they appear to focus too much on the other, that is, to facilitate the *oscillation* process that is central to the DPM. In the same spirit, participants were asked to list five suggestions for adaptations that could be used to tailor the intervention. The first questionnaire included a description of the LIVIA grief intervention, including an overview of the topics that the intervention treats in ten modules.

A thematic analysis (Braun and Clarke, 2006) was conducted on the qualitative *brainstorming* responses of the first questionnaire. A coding scheme was constructed in a bottom-up fashion and employed to the data. Two researchers independently coded the data using this coding scheme and a third researcher was involved to resolve conflicts.

For the adaptations, the initial coding scheme included codes for strategies that can be employed to tailor an online intervention. Additional codes covered suggestions regarding user parameters for personalization and theoretical and therapeutic frameworks to consider. For the monitoring parameters, each suggested parameter was coded. During the construction of the coding scheme, codes for overarching monitoring categories emerged and were included in the coding. Based on the coding, the adaptation model was constructed and user parameters were extracted and grouped into the overarching parameter categories.

### 4.2 Results

**Fig. 2:**
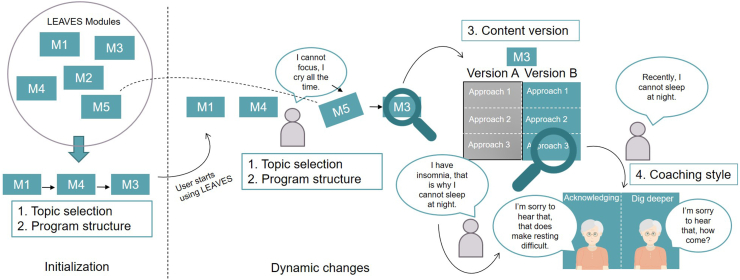
Visualization of the proposed adaptation model for personalizing the LEAVES intervention.

The two main outcomes of the first round of the Delphi study are the conceptual adaptation model depicted in Fig. 2 and a set of user parameters for monitoring purposes.

#### 4.2.1 Adaptations

The adaptation model consists of four types of adaptation strategies that can be employed to tailor LEAVES to the needs and characteristics of the user during the *initialization* of the service and *dynamically* while the user engages with the platform. The four types of adaptations are *Topic Selection*, *Program Structure*, *Content Version*, and *Coaching Style*. In the following, each adaptation type is presented in more detail, alongside some exemplary suggestions of the panelists that contributed to their conceptualization.

##### 4.2.1.1 Topic Selection

On the level of intervention modules, *Topic Selection* concerns *what* content is presented to the client. In contrast to the linear structure of the grief intervention LIVIA, a LEAVES user is presented with an individualized program based on an initial assessment. Subsequently, their individualized program is adjusted based on regular assessments as they progress through the intervention. Within *Topic Selection*, two adaptation strategies were proposed. First, the *removal of intervention modules* from the default configuration. Second, the *dynamic adjustment* of the selection of intervention modules based on regular re-assessment of the user's situation and progress. The latter strategy does include adding earlier removed intervention content to the user's personalized intervention program.

“Many online interventions are ‘hybrid’ meaning, that some modules are mandatory but others are optional dependent on the needs of the participant. For the LIVIA intervention, I expect that psycho-education and assessment of the current situation are mandatory modules that every participant should follow, but this may not be the case for all modules (e.g., not every participant may have problems with self-care or personal relationships).” (Participant 9)

##### 4.2.1.2 Program structure

On the level of intervention modules, *Program Structure* affects the structure of the client's personalized selection of therapeutic content. It determines *when* content is presented to the client, given a selection of intervention modules determined by the *Topic Selection* adaptation. Structural adaptations occur in the beginning of the LEAVES program as well as dynamically when the client is already using the program. Specific strategies that were proposed were *adjusting the order* of modules, *adjusting the length* of the intervention by manipulating the time spent on a topic and finally, *adjusting the length* by manipulating the number of modules or exercises on a topic.

“A possibility would be to suggest a priority list, and make participants begin the program by the factor that has been identified as the most salient or serious for each participant.” (Participant 11)

##### 4.2.1.3 Content version

On the level of individual intervention modules, the *Content Version* adaptation impacts *how* the content is presented to the user. In particular, two strategies for adjusting the content to the characteristics emerged from the suggestions. First, when the client appears to get stuck with the content of a module or an exercise, or they indicate that they want to try a different approach, the same content is presented from a different angle. An alternative approach to regular psycho education regarding a coping strategy could, for example, be a reflective exercise or a more playful “thought experiment” where the client tries out the benefits and consequences of the strategy first-hand in a type of adventure game. Another approach would be to identify concrete tasks and obstacles since the loss and to consider which resources in their daily life can be activated to address them:

“With regard to ‘unfinished business’ and ‘creating a new life’, the client may be guided to explicitly identify tasks that the deceased partner had performed and that now remain undone (e.g., managing the family finances, providing emotional support). In the next step, the client should specify how and by whom these tasks can be done now or the needs can be met now.” (Participant 2)

The second *Content Version* strategy is about re-writing the content of an intervention module depending on characteristics of the client. For example, the module about personal relationships could be better tailored by preparing a version for introvert versus more extrovert users.

##### 4.2.1.4 Coaching style

On the level of the conversations between the client and the embodied conversational agent (ECA) of LEAVES, *Coaching Style* concerns how the LEAVES program reacts to the input that the client provides. The adaptation encompasses strategies such as *acknowledging* hardship and reacting with appropriate empathy, including *personalized feedback* messages regarding regular monitoring assessments. Another strategy is keeping track of the story the client discloses to the system and to *point out changes or incoherencies* in the client's perceptions. A final strategy focuses on *interweaving* the input the user provides regarding different topics. For example, the ECA could build a conversation around what the user discloses regarding their hobbies and suggestions about how they can widen their social network. Regarding reflecting on changes in the discourse of the client, participant 12 suggested:

“Memories are also changing over the course of time and grief. It might be important to ‘store’ such memories and ask from time to time whether these memories still have the same quality. I could imagine a program that ‘reflects’ on personal memories in exchange with adaptations from the client.” (Participant 12)

#### 4.2.2 Monitoring parameters

On the basis of the brainstorming phase, we extracted 18 monitoring parameters for the construction of a user profile that informs decision making about professional support on-demand in LEAVES. The parameters were subdivided into four categories: *clinical*, *behavioral/emotional*, *interaction*, and *external*. Clinical parameters included symptoms that frequently occur in mourners, such as depressive and complicated grief symptoms. The behavioral/emotional category summarizes relevant user behaviors, such as the extent to which they are functionally autonomous, and relevant user characteristics such as the extent to which they are able to look ahead in the future positively. Both represent frequent risk factors for deterioration in (older) adult mourners. The interaction category includes parameters that describe the interaction of the user with the LEAVES service. Finally, the external parameter category encompasses two parameters that involve either events or people in the physical world outside the LEAVES intervention. Table 3 shows the 18 parameters that were extracted from the brainstorming phase. Variations in how the experts formulated their suggested monitoring parameters were treated in questionnaire two when the experts were able to give feedback on the completeness and appropriateness of the extracted parameters. For instance, the following three expert suggestions contributed to the definition of the *social isolation* monitoring parameter:

“If a client shows an increase in social avoidance or a reduction or complete lack of social contacts, blended treatment should be favoured.” (Participant 10)

“Ability to focus and derive comfort from other relationships (e.g., grandchildren).” (Participant 15)

“It could also be good to assess isolation or withdrawal behaviors, that is if the participant does not follow the program anymore because they are withdrawing from any activity or contact.” (Participant 11)

### 4.3 Concluding remarks

In the first round of the Delphi study, we elicited ideas for adapting the LEAVES intervention to the needs and characteristics of the user and parameters that can form the user profile for providing support on-demand. The first round yielded a conceptual adaptation model for LEAVES and an initial set of 18 monitoring parameters.

## 5 Delphi round 2

### 5.1 Method

The aim of the second questionnaire was to validate the conceptual adaptation model and to confirm that we correctly understood the suggested user parameters for monitoring. For this, we presented the adaptation model to our expert panel and asked them to comment on the extent to which it represented their suggested adaptations. We also encouraged them to pose any questions or make any remarks they may have about the proposed model. Specifically, we provided a list of the adaptation types and strategies with their respective definitions. For each adaptation strategy, we included an example. We also provided a holistic visualization of the adaptation model that summarized the adaptations and how they interact with each other to tailor the LEAVES intervention.

Regarding the monitoring parameters, we presented the 18 parameters that we extracted from the first questionnaire alongside their definitions. The parameters were subdivided into their four overarching categories: clinical, behavioral/emotional, interactive and external. As for the adaptations, we asked our experts to review the parameters and to state how the list represented their input. We also encouraged them to add any parameters that we may have overseen. In terms of analysis, the research team reviewed the questions and remarks the panelists submitted as response to the second questionnaire.

### 5.2 Results

Overall, the conceptual adaptation model was received positively and the presented list of monitoring parameters turned out to be quite exhaustive. There were some confusions regarding components of the adaptation model. For instance, one participant believed that the *Content Version* adaptation type impacted the modality of the module and not the therapeutic content itself. They thought that *Content Version* was about whether audio or video were included in the user interface design. Minor changes to the description of the conceptual adaptation model and the monitoring parameters were performed as a result of the input we received in the second round. In addition, personalized clarifications and answers to questions posed by panlists were provided to achieve a solid common understanding of the adaptation model and the monitoring parameters.

### 5.3 Concluding remarks

In the second round of the Delphi, we established common ground regarding terminology and a common understanding of the components of the adaptation model and the monitoring parameters. This round represented a necessary intermediate step towards a well-informed and reliable weighting of the components of the adaptation model and the monitoring parameters.

## 6 Delphi round 3

### 6.1 Method

The third questionnaire aimed to yield a weighting of the adaptations and parameters for monitoring. Given the abstract nature of the adaptation model defined in terms of personalization concepts, we chose to ask our mostly clinical expert panel for a *rating* of the adaptation strategies according to their perceived contribution to clinical outcome and to *rank* the overarching adaptation types. All adaptation strategies were rated on a 6-point Likert scale ranging from 1 (Not beneficial, even risky) to 6 (Extremely beneficial).

For the monitoring parameters, we asked the expert panel to rank the 18 parameters in three ways: By ranking the four parameter categories, by ranking all parameters within each parameter category, and finally, by selecting a top five most important parameters for decision making regarding escalation, regardless of their category. For all rankings, the order in which the monitoring parameters were presented was randomized for each panelist. We asked the panelist to briefly explain their rationale for the *rating* of the adaptations and the *ranking* of the parameters for monitoring.

The results of the third Delphi round were analyzed as follows. For both, the adaptations and the monitoring parameters, the median rating/rank was determined and the interquartile range (IQR) was calculated as a measure of dispersion. The IQR consists of the middle 50% of the observations and therefore, an IQR of less than 1 means that more than 50% of all rankings or ratings fall within 1 point on the scale. The IQR is frequently used in Delphi studies and it is generally accepted as an objective and rigorous method for assessing consensus (von der Gracht, 2012). For the top five selection, we calculated a ranking score. The score was a combination of the number of times a parameter was assigned a specific rank in the top five and a weight that was assigned to the rank, normalized by the size of the sample:

$$\frac{\sum_{i=1}^{5}x_{i}w_{i}}{N};\;w\in \left\{20,16,12,8,4\right\}$$

Where *x*_*i*_ is the count of how many times a parameter was ranked as rank *i*, *w*_*i*_ is the assigned weight to rank *i* and *N* is the sample size of the expert panel in the third round of the Delphi, *N* = 11. Adjusting the rank weights boiled down to balancing two values, the importance one assigns to being included in the top five selection versus the importance one assigns to a specific rank. We experimented with a number of weights and settled with *w* ∈ {20,16,12,8,4} because a) we considered being chosen as one of the top five parameters out of 18 too important to let individual ranks dominate the ranking and b) the order of the parameters ranked by score stayed stable. For the ranking of the parameter categories and the ranking of the behavioral/emotional parameters, two participants refrained from participating in the ranking, one participant for each ranking. In both cases, the participant felt that they lacked the required expertise for this ranking task.

### 6.2 Results

#### 6.2.1 Adaptations

Table 2 summarizes the ranking of the four adaptation types and the ratings for the eleven adaptation strategies. The following ranking order ranked highest to lowest can be extracted: Topic Selection, Program Structure, Content Version, and Coaching Style. Using the rule of thumb advocated by von der Gracht (von der Gracht, 2012) when using the inter-quartile range as a measure of consensus, the rankings for *Topic Selection* and *Program Structure* achieved reasonable consensus (*IQR* ≤ 1), and there was less agreement on the other two adaptations (*IQR* ≥ 1), *Content Version* and *Coaching Style*. For the adaptation strategies, scores were generally positive as all suggested strategies had a median rating of at least 3, *Somewhat beneficial*, five strategies had a median rating of 4, *Beneficial* and four a median rating of 5, *Very beneficial*. Regarding panelist agreement, three strategies were rated with good agreement (*IQR* ≤ 1) (*Adjusting the order*, *Different versions*, and *Acknowledging*) and six adaptations with reasonable agreement (*IQR* ≤ 1.5). The greatest divergence in expert opinion was obtained for *Adjusting the length (time)* of the intervention by manipulating the amount of time the user spends on a topic. Participant 12 is in favor of manipulating the time a user is advised to spend on a topic; Participant 8 is doubtful:

“It is an excellent idea to play with time and shape the intervention accordingly. Especially, I like the idea to give the client more exercises when needed.” (Participant 12)

“The suggested interpretation of ‘clicking through the program’ here might be too small: it might be the client's normal learning need to behave like this to answer a need to get an overview of what is available (understand the modules before determining a sequence). In such cases, a suggestion to slow down is not helpful to this client.” (Participant 8)

**Fig. 3:**
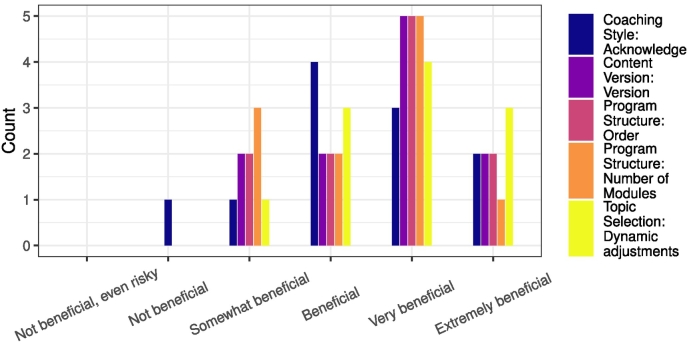
Bar chart of adaptation strategy ratings with comparable median and IQR ratings.

Fig. 3 shows how often the five highest rated adaptation strategies received which rating, regardless of their overarching adaptation category. The selection of the five most promising strategies was based on their median rating and IQR. Ratings were closely tied, but based on the counts, strategies *Dynamic adjustments* to the selected topics for a user's personalized program, dynamically manipulating the *Order* of intervention modules, and offering *Different versions* of the intervention content were rated highest.

**Table 2:** Median rank and inter-quartile range (IQR) for the four adaptation types. Median rating and IQR for the eleven adaptation strategies.

Adaptation **Type**/Strategy	Median **Rank**/Rating	IQR
**Topic Selection**	**2.0**	**1.0**
Dynamically changing topics	5.0	1.5
Removing from default	4.0	1.5
**Program Structure**	**3.0**	**1.0**
Adjusting the order	5.0	1.0
Overall length (n modules)	5.0	1.5
Overall length (time)	3.0	2.0
**Content Version**	**3.0**	**1.5**
Different versions	5.0	1.0
Repertoire of approaches	4.0	1.5
**Coaching Style**	**3.0**	**2.5**
Acknowledging	4.0	1.0
Personalized feedback messages	4.0	1.5
Reacting to incoherencies	4.0	1.5
Interweaving input	3.0	1.0

#### 6.2.2 Monitoring parameters

**Fig. 4:**
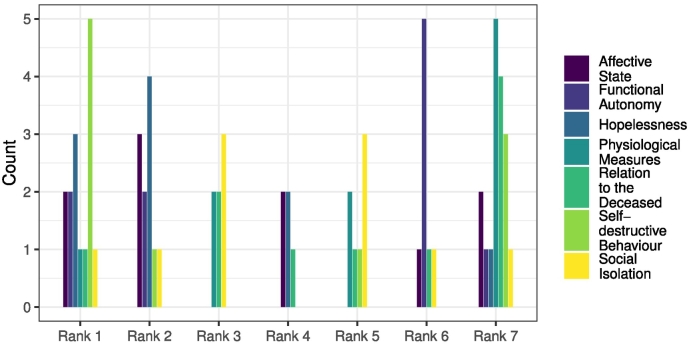
Bar chart of behavioral/emotional user parameter ranks. Counts are based on the responses of ten experts.

Table 3 summarizes the ranking for the four user parameter categories (*clinical*, *behavioral/ emotional*, *interaction*, and *external*), the intra-category ranking of individual user parameters and their calculated rank score. The ranking of the four monitoring parameter categories yielded a clear propensity towards the clinical parameters. Overall, consensus regarding parameter category ranks was good (*IQR* ≤ 1). Regarding intra-category rankings, for the clinical parameter category, *Suicidality* was ranked unanimously as the most important parameter for escalation, followed by *(Complicated) Grief*, *Depressive* and *PTSD* symptoms. With the exception of the latter parameter, there was good agreement regarding the ranking of each clinical parameter (*IQR* ≤ 1). The behavioral/emotional parameter category exhibited a less conclusive ranking. While having obtained the highest median rank, *Self-destructive behavior* exhibited the largest dispersion of assigned ranks. Based on the median rank and IQR, *Hopelessness* was ranked highest of all behavioral/emotional parameters for escalation, albeit with considerable disagreement (*IQR* ≤ 2). Fig. 4 shows how often each behavioral/emotional parameter was assigned which rank in the intra-category ranking. It appears that ranking *Self-destructive behavior* and *Functional Autonomy* had a polarizing effect. A subset of panelists ranked these two parameters high, while another subset ranked them low. *Hopelessness* was the only parameter with a trend towards higher ranks.

**Table 3:** Median rank and inter-quartile range (IQR) for the four parameter categories, the intra-category ranking and calculated ranking score for each parameter. *Median and IQR values based on the depicted number of experts.

	Median	IQR	Score
Parameter categories (*N* = 10*)			
Clinical	1.0	0.5	
Behavioral/Emotional	2.0	1.0	
Interaction	3.0	0.5	
External	4.0	1.0	

Clinical parameters			
Suicidality	1.0	0.0	15.64
(Complicated) Grief symptoms	2.0	1.0	5.45
Depressive sypmtoms	3.0	1.0	2.91
PTSD symptoms	3.0	1.75	3.27

Behavioral Emotional parameters (N = 10*)			
Hopelessness	2.0	2.25	4.73
Self-destructive behavior	1.5	4.5	6.55
Social isolation	3.5	1.0	0.73
Affective state	3.5	1.75	0.36
Functional autonomy	5.0	2.0	1.45
Physiological	6.5	1.75	0
Relation to the deceased	5.5	3.75	0

Interaction parameters			
Client-initiated escalation	2.0	1.0	6.18
Unresponsiveness	2.0	2.0	5.82
Coherence of discourse	3.0	2.0	1.09
Defence mechanisms	4.0	1.0	0
Too many questions	4.0	2.0	0

External parameters			
Events inducing vulnerability	1.0	1.0	2.91
Peer assessment	2.0	1.0	2.91

The ranking of the five interaction parameters was more conclusive, albeit with considerable disagreement regarding the rank of three out of five parameters (*IQR* = 2). There was good agreement regarding *Client-initiated escalation* and *Defence mechanisms* (*IQR* = 1). Regarding the final parameter category, external parameters, there was no clear preference for either external parameter. In sum, based on the three-fold ranking, the five highest ranked monitoring parameters are *Suicidality*, *Self-destructive behavior*, *Client-initiated escalation*, *Unresponsiveness*, and *(Complicated) Grief symptoms*, closely followed by the behavioral/emotional parameter *Hopelessness*. However, there is considerable disagreement regarding the importance of *Self-destructive behavior*, *Unresponsiveness* and *Hopelessness* when these parameters are ranked against other parameters within the same category.

### 6.3 Concluding remarks

The third round of the Delphi, yielded a *rating* of the components of the proposed adaptation model according to their expected capacity to increase clinical effectiveness and a *ranking* of the monitoring parameters according to their importance for decision making regarding professional support on-demand. There was considerable disagreement among panelists which emphasizes the importance of qualitative accounts of the panelists' rationale in addition to ratings and rankings in the Delphi process.

## 7 Discussion

This article describes the process and the results of a three-round Delphi study involving 16 grief and e-health experts to determine strategies for adapting an online grief intervention for older adults who lost their spouse and to identify parameters for a user profile for decision making regarding support on-demand. The Delphi study yielded a conceptual adaptation model whose components were rated by the expert panel according to their potential for increasing the clinical effectiveness of a text-based intervention. A preference emerged for dynamic *Topic Selection* and *Program Structure* adjustments as well as the possibility to offer tailored versions of the therapeutic text-based content. In contrast, adaptations that impact the *Coaching Style* of an embodied virtual agent (ECA) that guides the user through the intervention were received with scepticism regarding technical feasibility and health risks if not employed with utmost caution. A set of 18 monitoring parameters was elicited out of which *Suicidality*, *Self-destructive behavior*, *Client-initiated escalation*, *Unresponsiveness*, *(Complicated) Grief symptoms* and *Hopelessness* were ranked as most important to attend to for decision making regarding the intensity of professional support outside the online self-help service.

Two types of adaptations emerged from the suggestions of our expert panel: adaptations that impact the configuration of the service at initialization based on an initial assessment and dynamic adaptations that continue to re-adjust the intervention to the changing needs and preferences of the user. In a meta-analysis of tailored interventions for health behavior change, Krebs, Prochaska and Rossi (Krebs et al., 2010) found *dynamic tailoring* (assessing intervention parameters prior to each feedback provided to the user) to outperform *static tailoring* (basing all intervention feedback on one baseline assessment) regarding long-term intervention effect. While the effects for both, *static* and *dynamic* tailoring decrease over time, the authors found *dynamic tailoring* to still be statistically effective at twelve months after the intervention and attributed this to the enhanced relevance of feedback that reflects a person's change. This strengthens our panel's advice to focus on *dynamic* adjustments to enhance clinical effectiveness of the intervention. Regarding the user profile for delivering professional support on-demand, the panel's strong preference for clinical parameters can be attributed to the predominantly (clinical) grief expertise in the panel and the unanimous high ranking of *Suicidality* by it being life-threatening to the user and requiring immediate professional intervention, if present.

We were able to brainstorm ideas for adaptation and elicit monitoring parameters in a sample of 16 experts working in academia and clinical practice across four countries and to give a weighting to the elicited ideas to guide our future research efforts. The Delphi approach has been used at later stages of the development of personalized systems, for example, in rehabilitation to match recommendations regarding physical activity to the user's capabilities (Straat et al., 2020). However, the Delphi approach is rarely used in e-health research according to a recent review of human-centred methods for e-health development (Kip et al., 2022). The authors highlight two challenges for conducting Delphis in e-health. First, the recruitment of experts can be challenging because experts may be scarce in a new or specific field and regarding topics that involve novel applications of e-health technology. Second, reaching consensus can be time-consuming and complex. This paper shows how practical design choices can address these challenges when using the Delphi approach in early-stage personalization research for (mental) e-health. Regarding the first challenge, we chose to recruit an interdisciplinary expert panel including experts in grief and e-health instead of focusing on finding participants that possess expertise in both fields. Since we did not require a specific level of consensus as a stopping criterion and employed a fixed number of Delphi rounds instead, we limited the impact of consensus-seeking on the time effort required of the participants and the research team. The trade off between time and monetary investments and the extent to which consensus can be achieved in Delphis has been acknowledged (Hasson et al., 2000; von der Gracht, 2012).

Regarding the first strategy, recruiting an interdisciplinary expert panel, our subsequent choice to treat the experts as a single panel had implications for the level of detail of our results. We exposed grief experts to concepts from personalization and user profiling and subsequently asked them to rate these concepts. We also asked e-health experts to consider specific characteristics of mourners. In both cases, the experts in this study had to transfer their own domain knowledge. To maintain the accessibility of the adaptation model to all panelists, it was formulated in generic personalization terms and requires a considerable specification for the development of the LEAVES grief intervention. This constitutes a limitation of the chosen strategy. However, it does increase the transferablility of the resulting adaptation model to other mental e-health interventions for which a personalization strategy needs to be determined.

The latter strategy, refraining from using consensus in the rating and ranking tasks as stopping criterion and pre-determining the number of Delphi iterations instead, constitutes a deviation from Schmidt (Schmidt, 1997) and Okoli and Pawlowski (Okoli and Pawlowski, 2004) whose Delphi approach otherwise guided our study design. The common focus on reaching consensus in ranking-type Delphis has been criticized before for producing artificial consensus results and being an inadequate stopping criterion by itself (Dajani et al., 1979; von der Gracht, 2012). The current study endorses Gracht's statement about the primary goal of the Delphi approach: “the efficient structuring of a group communication process” (von der Gracht, 2012, page 1527). Pre-determining the number of Delphi iterations implied that consensus would be difficult to achieve in the rating and ranking tasks. There was indeed considerable disagreement regarding the rating of some adaptations and user parameters for monitoring.

Consequently, a limitation of the obtained rating and ranking results is that they should not be treated as an objective order of importance based on expert consensus, but rather as a starting point for subsequent efforts to specify a personlization strategy and for constructing a user profile to deliver support on-demand. Any follow-up efforts should scrutinize the suitability of highly rated adaptations and highly ranked user parameters for their specific purpose. Decision criteria that are relevant to their specific e-health application should be established. One criterion for determining the suitability of any monitoring parameter for any specific e-health application should be its sensitivity to changes in the user's situation (Gokalp and Clarke, 2013). The parameter's sensitivity must be compatible with the application's monitoring measurement interval to timely detect a deterioration of the user's situation.

Researchers who consider taking a Delphi approach for early-stage personalization research should consider the implications of the practical choices presented in this paper to address common challenges of Delphi studies in e-health. Specifically, if establishing consensus is desired in a Delphi approach with pre-determined iterations, the research team may consider replacing the brainstorming phase with pre-determined statements (e.g., Yap et al., 2017). When the number of iterations is not pre-determined, a hierarchical stopping criterion combining measures of group response stability over the course of several rounds and a consensus measure such as the interquartile range should be considered, as advocated by Schmidt (Schmidt, 1997) and Okoli and Pawlowski (Okoli and Pawlowski, 2004).

In conclusion, this study set out to determine a personalization strategy for an online grief intervention targeted at older adults who lost their spouse and to identify parameters for a user profile for delivering blended professional support on-demand. A conceptual adaptation model was constructed. Based on the ratings of eleven grief and e-health experts, *dynamic* adjustments, informed by regular assessment of user characteristics, to the selection of intervention topics and the order in which topics are presented emerged to be a promising personalization strategy. Indicators that capture perceived danger for the client and their ability to continue the intervention by themselves should be included in the user profile, including measures of *Suicidality*, *Self-destructive behavior* and *Client-initiated escalation*, *Unresponsiveness* and *(Complicated) Grief symptoms*. Based on our experiences from this study, the Delphi approach can be a useful early-stage research tool for exploring a personalization strategy for mental e-health interventions and for unraveling user parameters for decision-making about providing professional support on-demand.

## Declaration of competing interest

The authors declare that they have no known competing financial interests or personal relationships that could have appeared to influence the work reported in this paper.
